# Efficient Excitonic Configuration Interaction for Large-Scale Multichromophoric Systems Using the Resolution-of-Identity Approximation

**DOI:** 10.1021/acs.jpclett.5c00065

**Published:** 2025-03-10

**Authors:** Tomislav Piteša, Sebastian Mai, Leticia González

**Affiliations:** † Ruđer Bošković Institute, Bijenička cesta 54, 10000 Zagreb, Croatia; ‡ Institute of Theoretical Chemistry, Faculty of Chemistry, University of Vienna, Währinger Straße 17, 1090 Vienna, Austria; ¶ Research Platform on Accelerating Photoreaction Discovery (ViRAPID), University of Vienna, Währinger Straße 17, 1090 Vienna, Austria

## Abstract

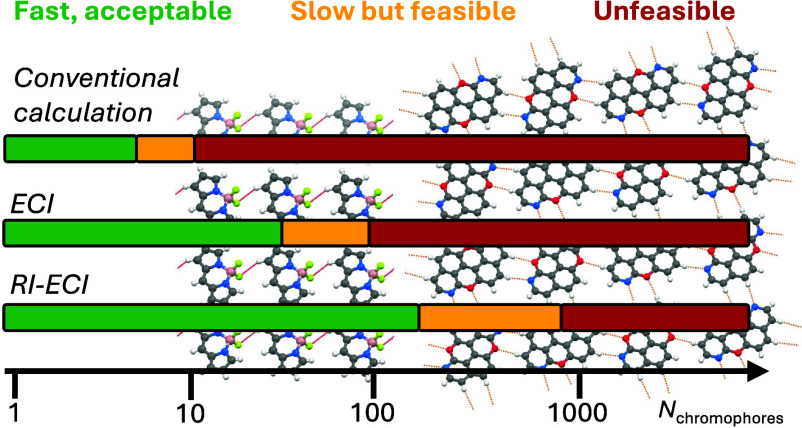

The calculation of electronic excited states in extended multichromophoric systems is computationally challenging. Here, we accelerate our recently introduced excitonic configuration interaction (ECI) method [T. Piteša et al. *J. Chem. Theory Comput.***2024**, 20, 5609] with the resolution-of-identity approximation for the two-site two-electron integrals in the calculation of the interchromophoric Coulomb and exchange terms. Additionally, a simple overlap-based scheme is introduced to prescreen the Cholesky-transformed tensor of the three-centric two-electron interchromophoric exchange integrals, significantly accelerating the expensive tensor contraction for the two-site exchange term. This reduces both cost and memory requirements, enabling large-scale calculations of systems with many chromophores. We demonstrate its efficiency and accuracy by calculating electronic excited states of chains of up to 32 BODIPY chromophores and networks of up to 100 peri-xanthenoxanthene units, with 12 320 and 43 600 basis functions, respectively. We achieve errors in the excitation energies below 30 meV, using site states calculated with time-dependent density functional theory.

The calculation of electronic excited states in extended multichromophoric systems using excitonic models has gained significant importance over the past couple of decades.^1−8^ They owe this popularity to their lower computational cost compared to direct (conventional) quantum-chemistry calculation. Some exciton models use the states of only individual chromophores,^2,3,5,7,9^ while others additionally use the states of chromophoric dimers, trimers, etc.^4,8^ In the former case, a typical exciton calculation of a system with *M* fragments consist of three steps: (i) the calculation of the electronic states of the individual *M* chromophores (the site-state calculations), (ii) building an excitonic basis of the antisymmetrized products of the site states, and (iii) evaluating and diagonalizing the exciton Hamiltonian in this excitonic basis. Since the electronic-structure calculations of the different fragments are independent, step (i) naturally scales with . In step (iii), many exciton models^2,3,5,7,9^ evaluate the exciton Hamiltonian within the so-called “strong-orthogonality approximation”,^10,11^ referring to the orthogonality of the orbitals originating from different sites and leading to expressions involving terms related to only pairs of fragments. Thus, in this case, step (iii) scales with . Further, although step (iii) scales quadratically, it exhibits a small prefactor and thus dominates only for very large numbers of fragments. For this reason, exciton calculations are, in principle, much faster and more appealing than direct full-system calculations, which usually scale as , where *n* > 2 depends on the employed quantum-chemistry method, or even exponentially ().

Recently, we presented the excitonic configuration interaction (ECI) method,^9^ which employs the exciton philosophy, but can, along with the ground-state (GS) product and Frenkel-like local excitations (LEs), describe multilocal excitations, i.e., configurations in which multiple sites are excited (DLEs: double-local excitations, TLEs: triple-local excitations, etc.). The ECI method does not only account for the couplings between two LEs on different fragments (as is common within the Frenkel model^12,13^), but employs the formally exact Hamiltonian within the strong-orthogonality assumption.^11^ This gives rise to the couplings between, e.g., different LEs on the same site, LEs and DLEs, etc., all of which are usually neglected in conventional exciton models, but which are important for the method to be systematically improvable.^9^ Further, the ECI method can be easily combined with any electronic-structure flavor because to build an ECI Hamiltonian, only site energies and site-specific one-particle density matrices are needed (vide infra). Finally, the ECI method can be combined with an embedding scheme with arbitrary embedding point charges,^9^ which can be constructed self-consistently so that the energy of the GS product is minimized (so-called “excitonic Hartree–Fock” (EHF)).

The construction of the ECI Hamiltonian (see ref (9) for further details) requires the calculation of the two-site Coulomb and exchange terms:
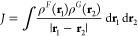
1
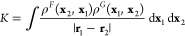
2where ρ^*F*^(**r**) and ρ^*F*^(**x**_1_, **x**_2_) are the one-particle state density and density matrix of a site state (or a transition density/density matrix between a pair of site states) of fragment *F*, and correspondingly for fragment *G*. When the one-particle densities are expressed in the basis of products of atomic orbitals (AOs), the terms become
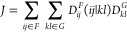
3
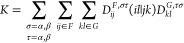
4where *D*^*F*^ is the matrix of the total density-matrix coefficients, *D*^*F*,στ^ is the matrix of the partial density-matrix coefficients, and (*ij*|*kl*) and (*il*|*jk*) are two-site two-electron integrals in the AO basis. In general, each fragment does not have a single but rather a number *N*_tot_^*F*^ of different total densities and *N*_part_^*F*^ different partial densities (depending on the number of site states) that, in combination with densities of another fragment, give rise to all *J* and *K* terms that need to be calculated by eqs 3 and 4 to construct the ECI Hamiltonian. Regardless of the involved density pair, the same two types of two-site two-electron integrals are needed—the first having a product of two AOs of the first fragment (*ij*) interacting with a product of two AOs of the second fragment (*kl*), and the other one having the product of an AO from *F* and an AO from *G* (*il*) interacting with a second product of the same kind (*jk*). These two types of two-electron integrals describe the Coulomb interaction and exchange between the fragments, respectively.

As the calculation of the two-electron integrals is expensive and requires a great amount of memory in an ab initio context (i.e., without semiempirical schemes), this Letter presents an application of the resolution-of-identity (RI) scheme^14−17^ to reduce both the time and memory demands of building the ECI Hamiltonian. This scheme does not reduce the scaling of step (iii) with *M*, but significantly cuts down its prefactor and hence makes step (iii) become more expensive than step (i) only for very large *M*. Furthermore, we employ a simple integral-prescreening scheme to neglect small integrals contributing to the *K* terms, which does cut down the quadratic scaling of step (iii). Finally, we illustrate the error of the introduced approximations and the performance of the RI-ECI method on two examples, a linear chain and a two-dimensional network of organic chromophores.

In detail, within the RI approximation, the two-electron integrals needed in ECI are approximated as
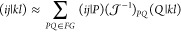
5
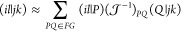
6where *P* and *Q* are both auxiliary basis functions of the *F*–*G* complex, (*ij*|*P*) and similar terms are three-centric two-electron integrals, and  is the matrix of the two-centric two-electron integrals in the auxiliary basis. As, AOs *i* and *j* originate from the site *F*, while AOs *k* and *l* originate from the site *G*. In our implementation, all four types of electron-repulsion integrals, (*ij*|*P*), (*kl*|*Q*), (*il*|*P*) and , are calculated with the libcint package^18^ interfaced through PySCF.^19^ In this computational framework, (*ij*|*P*) and (*kl*|*Q*) are elements of two different 3D tensors, while (*il*|*P*) and (*jk*|*Q*) are just different elements of a single 3D tensor. Hence, in addition to a 2D tensor  of the size (*N*_aux_^*FG*^)^2^ common for both *J* and *K* terms, for the *J* terms, one needs to store two tensors of the total size [(*N*_AO_^*F*^)^2^ + (*N*_AO_^*G*^)^2^] *N*_aux_^*FG*^, while for the *K* terms, one needs to store a single tensor of the size *N*_AO_^*F*^*N*_AO_^*G*^*N*_aux_^*FG*^. This is usually much lower than the memory requirements of canonical ECI, which for a pair of fragments demands storage of two 4D tensors of total size 2(*N*_AO_^*F*^)^2^(*N*_AO_^*G*^)^2^. Of course, memory demands of both canonical ECI and RI-ECI could be reduced by an implementation in which, e.g., not whole tensors are calculated at once but rather in slices (per one or more dimensions) and contracted with the corresponding density-matrix coefficients in series.

The *J* and *K* terms within the RI approximation are then
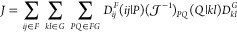
7
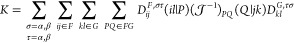
8The *J* terms can be efficiently calculated by a series of matrix-multiplication-like tensor contractions along one or two axes. In particular, an effective tensor contraction sequence for most use cases is *fij*, *ijP* → *fP*, followed by *fP*, *PQ* → *fQ*, followed by *fQ*, *Qkl* → *fkl*, followed by *fkl*, *g*(*f*)*kl* → *fg*(*f*), where index *f* runs over different *D*^*F*^s and *g*(*f*) run over different *D*^*G*^s for a given *f*. The biggest intermediate tensor of this contraction sequence is rather small, being of a size *N*_tot_^*F*^ (*N*_AO_^*G*^)^2^. On the other hand, an efficient path in the contraction for *K* terms is *fij*, *ilP* → *fjlP*, followed by *fjlP*, *PQ* → *fjlQ*, followed by *fjlQ*, *Qjk* → *flk*, followed by *flk*, *g*(*f*)*kl* → *fg*(*f*). As can be seen, this path involves large 4D intermediates in the first two steps (of a size *N*_part_^*F*^*N*_AO_^*F*^*N*_AO_^*G*^*N*_aux_^*FG*^), and requires swapping the axes in the last two steps to adjust the contiguity of the tensors for the fastest matrix multiplication. Hence, this contraction for *K* is much more demanding than the one for *J*. However, we found that in this case, Cholesky decomposition of the  matrix accelerates the overall contraction significantly. Here, a *K* term becomes
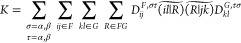
9where
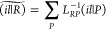
10with **L** being the lower triangular Cholesky-factor matrix of  (i.e., ). Note that the tensor  can be efficiently calculated by solving the generalized system of equations without explicitly calculating **L**^–1^, due to the fact that **L** is triangular. For the *J* terms, the contraction with Cholesky decomposition appears to be more expensive, because tensors (*ij*|*P*) and (*kl*|*Q*) are different and thus two generalized systems of equations equivalent to eq 10 would need to be solved.

For two sites that are far apart, the majority of *il* products in eq 9 for the *K* terms are practically zero functions; hence, many  are negligible. Thus, we first calculate the intersite AO-overlap matrix **S**^*FG*^ and extract only those AO shells *I* ∈ *F* and *L* ∈ *G* for which |*S*_*il*_^*FG*^| > *t*_*S*_, for any two *i* ∈ *I* and *l* ∈ *L* atomic orbitals, where *t*_*S*_ is an arbitrary threshold. Then we proceed to the calculation of  and contraction in eq 10 with only the extracted shells from both sites. We would like to stress that there are multiple other integral-prescreening schemes in the literature,^20^ many of them being more robust and reliable that this one. However, we opted for this simple overlap-based scheme for the sake of speed and ease of implementation, that should work equally well if the fragments are well-separated. This prescreening can drastically accelerate the calculation of the *K* terms, without significant loss of accuracy if an appropriate value of the threshold *t*_*S*_ is used, as we show below. Also, the addition of the prescreening scheme to the RI approximation cuts down the quadratic scaling of *K*-term calculations because for large *M*, the vast majority of the site pairs are going to be far apart and thus will yield no *il* AO pairs with significant overlap, i.e., many *K* terms are going to be completely neglected. Note, however, that intersite AO overlaps are generally expected to be small in the systems where ECI is applicable, as otherwise the strong orthogonality assumption would be likely violated. To additionally accelerate the tensor contractions in the *K*-term calculations, we delete slices of the calculated  tensor in all three dimensions wherever the absolute value of each element is lower than another threshold *t*_*C*_. The presented RI and prescreening schemes are implemented within a local development version of the SHARC package,^21,22^ on top of the canonical ECI implementation.^9^

The applicability of the RI-ECI approach is first exemplified on a chain of BODIPY (boron-dipyrromethene) dyes connected via C–H**···**F hydrogen bonds (Figure 1a), a motif found in its crystal structure.^23^ Using the experimental X-ray geometry, we carried out calculations for systems containing *M* = 1, 2, 4, 8 BODIPY molecules, computing *M* excited singlet and *M* triplet states using time-dependent density functional theory (TD-DFT). The level of theory was TD-ωB97X-D/may-cc-pVDZ^24,25^ within the Tamm–Dancoff approximation, as implemented in Gaussian 16.^26^ The results of the *direct full-system calculations* were used as reference data, against ECISD and RI-ECISD. In the site-state calculations, for each fragment the S_0_, S_1_ and T_1_ site states were computed at the same TD-ωB97X-D/may-cc-pVDZ level of theory. In the RI-ECISD calculations, the aug-cc-pVDZ-RI auxiliary basis set^27^ was used as described above (see eqs 7 and 9). We note that ECI is formally compatible with site states calculated at any level of theory, as the construction of the ECI Hamiltonian only requires site energies and site one-particle density matrices.^9^ Prior to the site-state calculations, we performed a self-consistent EHF computation to obtain embedding charges in whose field the site states are computed (convergence criterion *t*_*Q*_ = 10^–3^ for the change of the point charges^9^). The efficiency of our method in extended multichromophoric systems with many fragments is illustrated with (RI-)ECISD calculations for *M* = 16 and *M* = 32, for which direct TD-DFT calculations are computationally too demanding.

**Figure 1:**
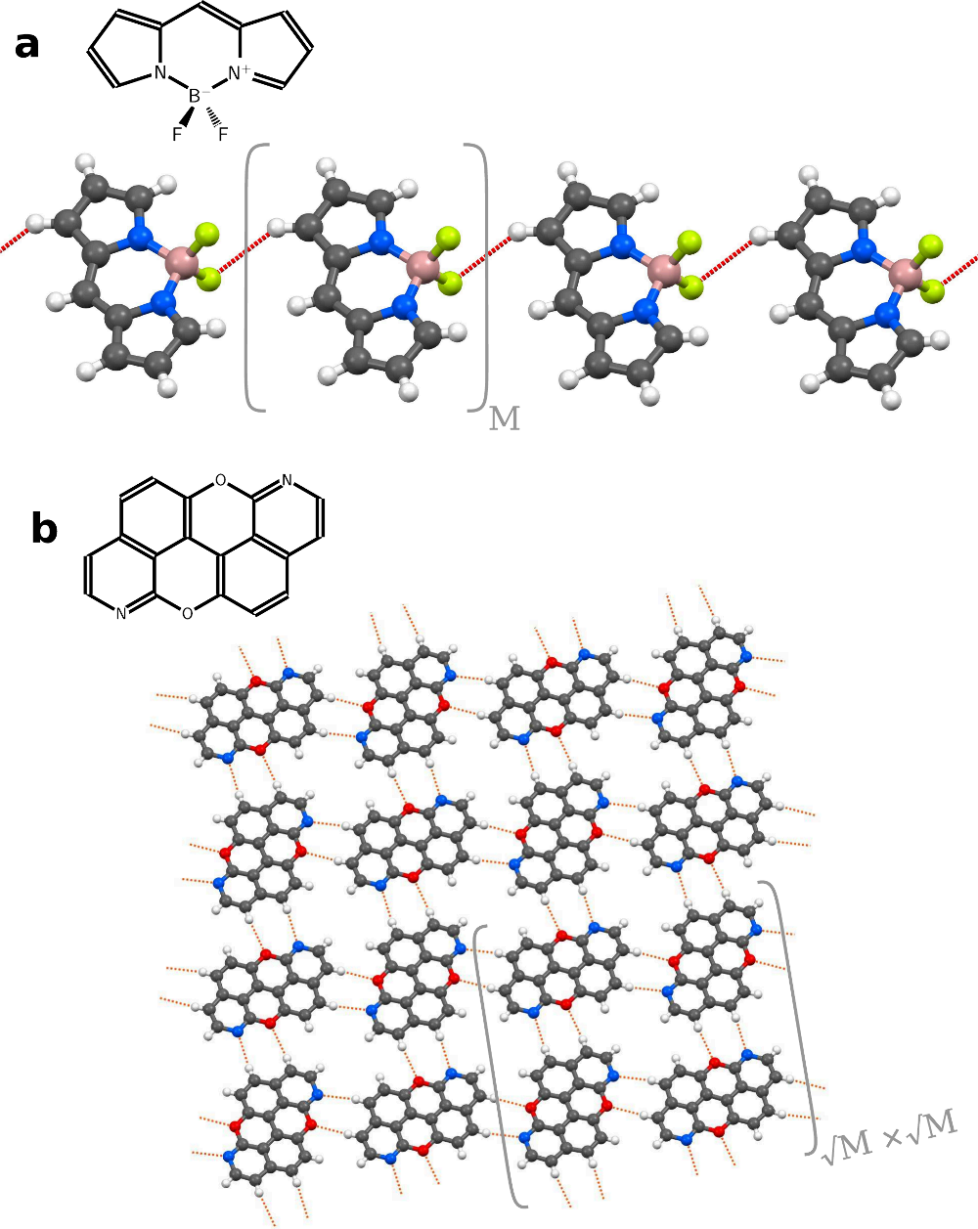
Molecular structure of (a) BODIPY and (b) PXXN molecule, and the supramolecular motifs calculated in this work from their respective crystal structures.

Given the employed ECISD expansion and the set of site states for each BODIPY, the ECI basis of BODIPY multimers includes the following singlet states: (i) the GS product ^1^**0̃**, (ii) *M* different S_1_ LEs ^1^**0̃**^*F*S_1_^, (iii)  different T_1_T_1_ DLEs ^1^**0̃**^*F*T_1_,*G*T_1_^, and (iv)  different S_1_S_1_ DLEs ^1^**0̃**^*F*S_1_,*G*S_1_^. Given that we diagonalize the entire ECI Hamiltonian, we compute (ii) + (iii) + (iv) = *M*^2^ full-system excited singlet states. The ECISD basis furthermore includes the following triplet states: (i) *M* different T_1_ LEs ^3^**0̃**^*F*T_1_^, (ii)  different T_1_T_1_ DLEs ^3^**0̃**^*F*T_1_,*G*T_1_^, (iii) and *M*(*M* – 1) different T_1_S_1_ DLEs ^3^**0̃**^*F*T_1_,*G*S_1_^, for a total of  states. The expansion of these spin-adapted configurations in terms of raw antisymmetrized products of the site states is given in Section S1 in the electronic Supporting Information (ESI). We emphasize that the direct TD-ωB97X-D calculation inherently describes only the LE configurations ^1^**0̃**^*F*S_1_^ and ^3^**0̃**^*F*T_1_^ due to its CIS-like nature. Thus, comparisons between (RI-)ECISD and TD-DFT are limited to states primarily described by LEs. States of dominant DLE character in the(RI-)ECISD calculations are discussed separately, without referencing TD-DFT.

Before analyzing the results, we comment on the errors of RI and the prescreening approximation on the *K* terms. First, for *M* = 2, we have independently scanned the prescreening thresholds *t*_*S*_ and *t*_*C*_ from 10^–6^ to 10^–2^ (Figure S1). We observe that the cheapest threshold combination that gives a maximum error of the *K* terms below 10 μHartree (0.3 meV) is *t*_*S*_ = 10^–4^ and *t*_*C*_ = 10^–3^. Hence, this combination was used in all subsequent RI-ECISD calculations on the BODIPY chains. Second, using the optimized combination of the prescreening thresholds, we analyze the overall error introduced by the RI approximation of the matrix elements of the ECI Hamiltonian, coming from both *J* and *K* terms (Figure S2). For the *J* terms, the RI approximation mostly introduces errors in the Hamiltonian diagonal matrix elements, found in the range of10–19 μHartrees (0.3–0.6 meV). The same trend can be observed for the *K* terms, with errors on the diagonal around −7 μHartrees (−0.2 meV). The errors of both *J* and *K* couplings (off-diagonal elements) are below 1 μHartree (0.03 meV). Hence, the errors in the total ECI Hamiltonian matrix elements are found in the range of 18–26 μHartrees (since *H* = *J* – *K*), or below 0.7 meV, while the total errors of the couplings stay below 1 μHartree (0.03 meV).

Having quantified the (small) errors of the presented RI/prescreening approach, we can now compare ECI/RI-ECI with the direct TD-DFT calculation of the systems with *M* = 1–8. Figure 2 compares the electronic absorption cross section^28^ and density of states (DOS) between the direct TD-DFT calculation (red) and RI-ECISD (dark blue) for each considered *M* (see Section S2 for details how the spectra and DOS are computed). Here, the cross section (bottom plots) shows an observable with only bright states visible, whereas the DOS (top plots) includes both bright and dark states. In agreement with the results of the error testing above, the differences between canonical ECISD and RI-ECISD are very small—excitation energies agree within 2 meV and oscillator strengths within 0.004 per molecule. The error of the absolute ground state energy (per site) was between −0.2 meV and +3 meV, with mean absolute error of 1 meV. Hence, for simplicity, Figure 2 only shows the RI-ECISD results; the ECISD values can be found in Figure S3.

**Figure 2:**
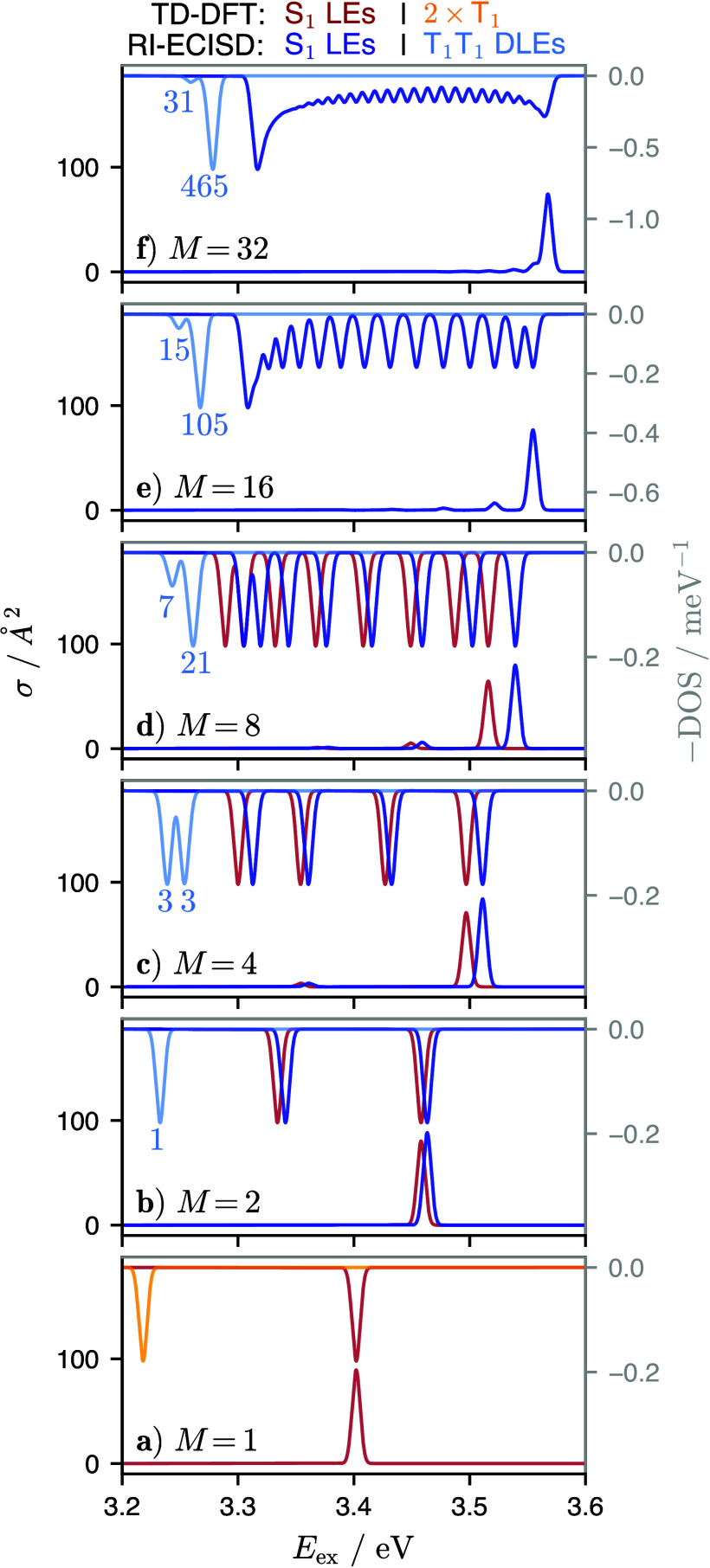
Molar absorption cross section σ (lower *x*-axis, left *y*-axis) and DOS (upper *x*-axis, right *y*-axis) for the segments of BODIPY chain containing *M* = 1, 2, 4, 8, 16, 32 fragments. Results obtained from a direct TD-DFT calculation are shown in red and RI-ECISD in dark blue, with δ*E* = 7 meV (see eq S10). Light blue curves display the DOS of dominantly TT states calculated by RI-ECISD and are scaled to match the maximum of DOS of S_1_ LEs. The numbers beneath the peaks of TT DOSes are the number of TT states in each peak in DOSes. Orange curve for *M* = 1 represent the position of doubled excitation energy of the T_1_ site state.

Figure 2a shows that the S_1_ state of a single BODIPY dye (*M* = 1), predicted at 3.40 eV, splits into two peaks in the BODIPY dimer (*M* = 2), with a 0.12 eV splitting (see DOS). As visible in the spectrum, only the upper excitonic state is bright, whereas the lower one is dark. RI-ECISD accurately reproduces the positions of those two peaks, with a deviation below 6 meV with respect to the direct TD-DFT calculation. As *M* increases, the set of S_1_ states splits further apart, with the highest state being always the brightest one. With increasing *M*, the error in the position of that bright peak increases, which we attribute to the lack of size extensivity of (truncated) ECI—the error observed for a single fragment pair accumulates with an increasing number of fragment pairs. Including additional site states and excitation ranks beyond ECISD could be used to alleviate such errors. Nevertheless, at RI-ECISD level, for *M* = 8 the error in the bright peak’s position is still only 23 meV. We also observe that bright and dark states occur alternatively, with the highest state being the brightest, and the third-highest, fifth-highest, etc., becoming progressively darker. When *M* increases to 16 and 32, the density of S_1_ LE states increases to a point in which the individual peaks start overlapping (with the employed Gaussian broadening) and hence form a contiguous band, characteristic of periodic excitonic systems; this is particularly visible in the DOS for M = 32. We thus demonstrate that RI-ECI has the potential to calculate systems sufficiently large so that bulk features are retrieved from a molecular quantum-chemistry approach.

Figure 2 also shows the behavior of the states dominantly described by T_1_T_1_ DLEs (“TT states” henceforth). As can be seen, a single TT state (for *M* = 2) is slightly blue-shifted with respect to twice the T_1_ excitation energy (shown in orange in *M* = 1). This is exclusively due to the somewhat less stabilizing interaction of two BODIPY molecules in T_1_ than in S_0_ site states. For *M* = 4, the TT band slightly splits due to exchange-driven couplings, and keeps getting blue-shifted. This leads to TT band coming closer to the band of S_1_ LEs and, judging by the trend observed until *M* = 32, it is reasonable to assume that the two bands are partially overlapping in the bulk. This is in accordance with the observed efficient singlet fission in BODIPY films.^29^

We note that our implementation of RI-ECI can calculate full-system states of arbitrary multiplicity, if appropriate site states are provided. For example, see the excellent agreement also obtained for the TD-DFT and RI-ECISD triplet states in Figure S4.

Figure 3 compares the CPU demand and scaling of the direct TD-DFT, ECISD, and RI-ECISD calculations on the BODIPY chains. The total TD-DFT calculation has an apparent scaling of *M*^*n*^, *n* = 2.46, resulting from a combination of DFT scaling with *n* = 2.10 and excited-state (TD-DFT) calculation scaling with *n* = 2.50. In contrast, canonical ECI (i.e., without RI and prescreening, red in Figure 3) overall scales with *n* = 1.62 and RI-ECI (blue) scales almost linearly with *n* = 1.07. Here, the shared EHF and site state calculation steps (purple) scale essentially linearly, as they consist of independent SCF and excited-state calculations. The excitonic part of canonical ECI scales nearly quadratically (*n* = 2.19) with a prefactor of *A* = 0.309, whereas for RI-ECI, the prefactor drops to 0.069 due to the RI approximation and the exponent drops to *n* = 1.78 due to the prescreening of the *K* terms. In essence, for the presented application, the overall subquadratic/linear scaling of ECI/RI-ECI is achieved as a combination of a linear-scaling part with large prefactor (EHF and site-state calculations) and a part with quadratic/subquadratic scaling and small prefactor (excitonic calculation). We note that the prefactors of EHF and the site-state calculations depend on the electronic-structure method employed for the sites, while the excitonic part is agnostic to the employed method (up to the employed basis set, see below). Hence, for expensive electronic structure methods (e.g., EOM-CC or CASPT2, but also TD-DFT), effective linear scaling can be achieved because the cost of the excitonic step is essentially negligible compared to the site-state calculations. In contrast, for inexpensive electronic structure methods (e.g., semiempirical methods or tight-binding models) the EHF and the site-state calculations will have small prefactors and thus the effective scaling of RI-ECI will shift toward the (subquadratic) scaling of the excitonic part of the calculation. A separate aspect is the scaling of RI-ECI with the size of each site, i.e., the number of AOs per site. The EHF and site-state calculations inherit the scaling of the employed electronic structure method with the number of AOs, and the site-state calculation additionally scales with the number of computed site states. The excitonic part of RI-ECI scales approximately as (*N*_AO_^*F*^)^2^*N*_aux_^*FG*^, assuming identical sites. Additionally, the timing of excitonic part depends on the number of site states and the employed ECI basis (ECIS, ECISD, ...).

**Figure 3:**
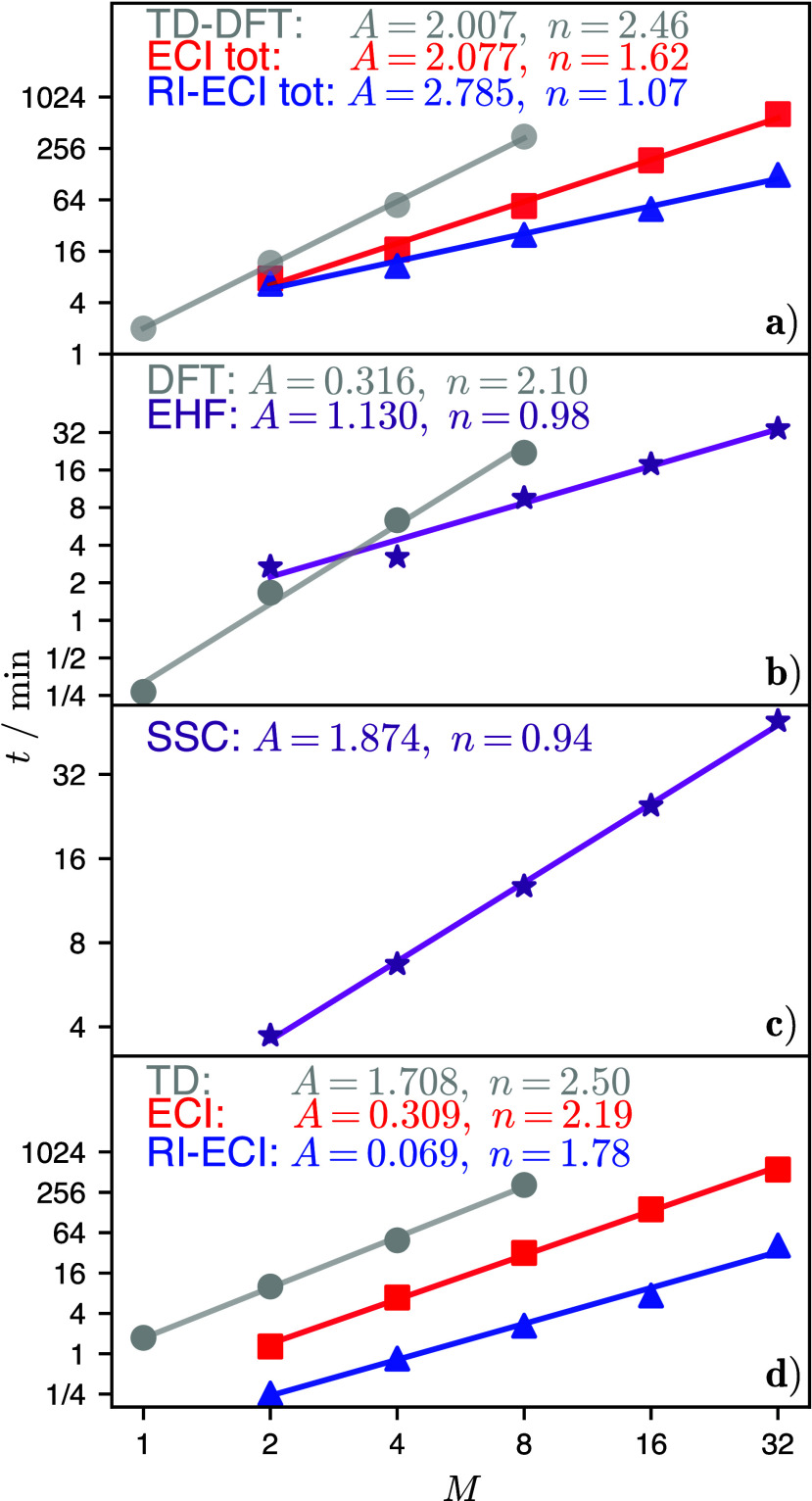
(a) Wall times of direct TD-DFT (gray), ECISD (red), and RI-ECISD (blue) calculations of BODIPY chains for different sizes *M*, on two AMD EPYC 7502 32-Core CPUs (64 cores total). (b, c, d) Wall times of different parts of the calculations. The direct TD-DFT calculation consists of the SCF (panel (b), gray) and TD (panel (d), gray) parts. The ECISD and RI-ECISD calculations share the EHF (panel (b), purple) and site-state calculations (SSC, panel (c), purple), but have different excitonic parts (panel (d), red/blue). All data are fitted to log (*t*/min) = log *A* + *n* log *M*, and both axes exhibit a logarithmic scale, so that the polynomial dependence of the wall time with the system size can be seen.

In the BODIPY system, all chromophores have the same orientation and thus their S_0_–S_1_ transition dipole moments (oriented along the long axis of each molecule) are aligned in parallel. Hence, this system can be seen as an H-aggregate with a strong exciton splitting (around 180 meV in bulk). Moreover, the molecules are assembled in a 1D structure, so each BODIPY has only one or two neighbors and thus strongly interacts only with a handful of other chromophores. Hence, the second showcase of our methodology is a supramolecular two-dimensional network of nitrogen-doped peri-xanthenoxanthene (PXXN) molecules,^30^ connected via C–H**···**N and C–H**···**O hydrogen bonds (Figure 1b). Various peri-xanthenoxanthene derivatives, as simple models of polycyclic aromatic hydrocarbons, have recently gained interest as light-harvesting compounds to be used in photocatalysis.^30^ In this system, the choromophores and S_1_ transition dipole moments (also oriented along the long axis) are not parallel for each pair of sites. Hence, the exciton splitting is expected to be much weaker than in the BODIPY chain. However, each PXXN molecule interacts directly with up to four neighbors, and hence the interaction energy terms between fragments (e.g., state-specific strengths of hydrogen bonds) are expected to be relevant. Square-like cuts containing *M* = 1 × 1, ..., 10 × 10 molecules are calculated with the same methodology as for BODIPY, except that only one cycle of EHF has been performed, an ECIS expansion was used, and tighter thresholds of *t*_*S*_ = 10^–8^ and *t*_*C*_ = 10^–7^ were employed (as discussed below). For the PXXN systems, only RI-ECI calculations are done (no canonical ECI) and only singlet states are calculated. Due to the ECIS expansion and using only one excited singlet per site, the number of excited full-system states is *M*.

Figure 4 shows the spectrum and DOS for all *M* values of the PXXN network. In the direct TD-DFT calculation of the isolated PXXN (*M* = 1 × 1) dye, there is one bright state slightly below 3.51 eV. When going to *M* = 2 × 2, the overall four S_1_ LE states are found to occur as two pairs of quasi-degenerate states (one dark and one bright), giving rise to two absorption peaks with a splitting of about 7.4 meV. The RI-ECIS calculation also predicts two absorption peaks (9 meV splitting), slightly blue-shifted (by 5 and 7 meV for lower and upper peak) compared to the direct calculation. We note that in the RI-ECI for *M* = 2 × 2, the dark states are not degenerate with the bright states, as evidenced by the third peak in the DOS. We assume that this deviation between direct calculation and RI-ECI arises because the energy splittings in this system are very small and of the same size as the expected errors of ECI, as reported for other systems of this kind.^9^ Additionally, the RI approximation and the prescreening procedure further amplify the error of the ECI calculation, making the deviations from the direct calculation very difficult to rationalize.

**Figure 4:**
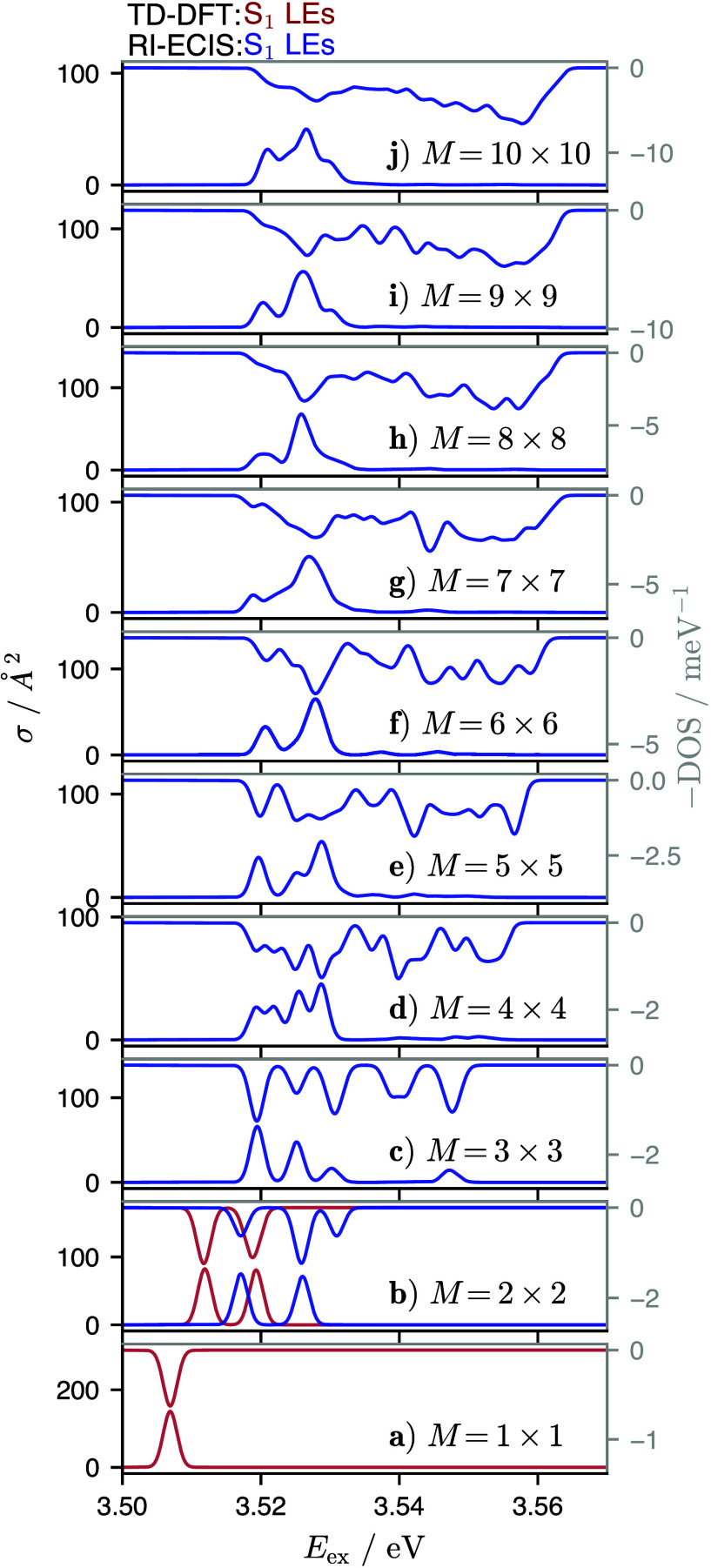
Molar absorption cross section σ (lower *x*-axis, left *y*-axis) and DOS (upper *x*-axis, right *y*-axis) for the square-like cuts of PXXN network containing *M* = 1 × 1, ..., 10 × 10 fragments. Results obtained from a direct TD-DFT calculation are shown in red and RI-ECIS in dark blue, with Gaussian broadning of δ*E* = 2 meV (see eq S6).

The splitting pattern and brightness of the full-system states depends critically on the linear combinations of LEs that describe these states. For the direct calculation of *M* = 2 × 2, the first pair of states is nearly , while the second one is , where site 1 is located diagonally opposite to site 4, and site 2 to site 3 (see Figure 1b). These linear combinations reflect the *C*_*i*_ symmetry of the system and give rise to the bright and dark full-system states. In the RI-ECI calculation, such linear combinations could only be obtained by using very tight prescreening thresholds of *t*_*S*_ = 10^–8^ and *t*_*C*_ = 10^–7^. If the thresholds are set more loosely, relevant excitonic coupling terms will be neglected and rather than linear combinations of LE states one obtains localized LE states, each of them being moderately bright. This shows that the prescreening thresholds need to be considered cautiously, especially for systems where intersite couplings are small, as illustrated in the PXXN test system.

Already for *M* = 3 × 3, a direct calculation cannot be completed within a week of wall time on our hardware (two AMD EPYC 7502 CPUs with 64 cores total). In contrast, the RI-ECIS calculation up to *M* = 10 × 10 took about 15 h of wall time (on 64 cores), out of which the exciton part took 52%. Similar to the BODIPY chain, Figure 4 shows how the spectrum and DOS evolves from line-like features for lower *M* values to continuous band-like ones for higher *M* values, reproducing bulk features (i.e., delocalized states) from calculations that are based on molecular (localized) building blocks.

In conclusion, we demonstrated that the RI approximation with exchange prescreening introduces minimal errors in both absolute and excitation energies of ECI, while significantly accelerating the calculation. The efficiency of this approach was illustrated by calculating the first 100 singlet excited states of a system containing 100 chromophores, each having 436 AOs, using TD-DFT-based site states. The calculation was completed in about 15 wall hours on 64 cores, with significantly reduced memory usage compared to canonical ECI. The overall scaling of RI-ECI with TD-DFT site states and EHF embedding was found to be nearly linear with the number of fragments. We also showed that the method provides a qualitatively correct description of the band-like structure of electronic spectra of 1D and 2D periodic motifs, and can be easily applied to 3D structures as well. As such, it offers a powerful tool for modeling stationary electronic properties of a wide range of extended multichromophoric systems, such as supramolecular aggregates, porous networks, and weakly bonded coordination polymers.

## Supporting Information Available

Expansion of singlet and triplet spin-adapted excitonic configurations of BODIPY in terms of raw antisymmetrized products. Analysis of the error introduced by RI and prescreening approximations to the ECI Hamiltonian matrix elements. Comparison of direct TD-DFT, ECISD and RI-ECISD states of the BODIPY chains. DOS of the triplet states of BODIPY chains (PDF)

## References

[ref1] Morrison A. F.; You Z. Q.; Herbert J. M. Ab initio implementation of the Frenkel-Davydov exciton model: A naturally parallelizable approach to computing collective excitations in crystals and aggregates. J. Chem. Theory Comput. 2014, 10, 5366–5376. 10.1021/ct500765m. 26583220

[ref2] Li X.; Parrish R. M.; Liu F.; Kokkila Schumacher S. I. L.; Martinez T. J. An Ab Initio Exciton Model Including Charge-Transfer Excited States. J. Chem. Theory Comput. 2017, 13, 3493–3504. 10.1021/acs.jctc.7b00171. 28617595

[ref3] Menger M. F.; Plasser F.; Mennucci B.; González L. Surface Hopping within an Exciton Picture. An Electrostatic Embedding Scheme. J. Chem. Theory Comput. 2018, 14, 6139–6148. 10.1021/acs.jctc.8b00763. 30299941

[ref4] Green J. A.; Asha H.; Santoro F.; Improta R. Excitonic Model for Strongly Coupled Multichromophoric Systems: The Electronic Circular Dichroism Spectra of Guanine Quadruplexes as Test Cases. J. Chem. Theory Comput. 2021, 17, 405–415. 10.1021/acs.jctc.0c01100. 33378185

[ref5] Gil E. S.; Granucci G.; Persico M. Surface Hopping Dynamics with the Frenkel Exciton Model in a Semiempirical Framework. J. Chem. Theory Comput. 2021, 17, 7373–7383. 10.1021/acs.jctc.1c00942. 34843643 PMC8675141

[ref6] Friedl C.; Fedorov D. G.; Renger T. Towards a quantitative description of excitonic couplings in photosynthetic pigment-protein complexes: Quantum chemistry driven multiscale approaches. Phys. Chem. Chem. Phys. 2022, 24, 5014–5038. 10.1039/D1CP03566E. 35142765 PMC8865841

[ref7] Kaiser A.; Daoud R. E.; Aquilante F.; Kühn O.; De Vico L.; Bokarev S. I. A Multiconfigurational Wave Function Implementation of the Frenkel Exciton Model for Molecular Aggregates. J. Chem. Theory Comput. 2023, 19, 2918–2928. 10.1021/acs.jctc.3c00185. 37115036

[ref8] Sangiogo Gil E.; Giustini A.; Accomasso D.; Granucci G. Excitonic Approach for Nonadiabatic Dynamics: Extending Beyond the Frenkel Exciton Model. J. Chem. Theory Comput. 2024, 20, 8437–8449. 10.1021/acs.jctc.4c00886. 39284746

[ref9] Piteša T.; Polonius S.; González L.; Mai S. Excitonic Configuration Interaction: Going Beyond the Frenkel Exciton Model. J. Chem. Theory and Comput. 2024, 20, 5609–5634. 10.1021/acs.jctc.4c00157. 38885637 PMC11238547

[ref10] McWeeny R. The density matrix in many-electron quantum mechanics I. Generalized product functions. Factorization and physical interpretation of the density matrices. Proc. R. Soc. A 1959, 253, 242–259. 10.1098/rspa.1959.0191.

[ref11] McWeeny R. Methods of Molecular Quantum Mechanics, 2nd Edition; Academic Press: London, 1992.

[ref12] Frenkel J. On the Transformation of Light into Heat in Solids. I. Phys. Rev. 1931, 37, 17–44. 10.1103/PhysRev.37.17.

[ref13] Davydov A. S. The Theory of Molecular Excitons. Sov. Phys. Usp. 1964, 7, 145–178. 10.1070/PU1964v007n02ABEH003659.

[ref14] Feyereisen M.; Fitzgerald G.; Komornicki A. Use of approximate integrals in ab initio theory. An application in MP2 energy calculations. Chem. Phys. Lett. 1993, 208, 359–363. 10.1016/0009-2614(93)87156-W.

[ref15] Jung Y.; Sodt A.; Gill P. M. W.; Head-Gordon M. Auxiliary basis expansions for large-scale electronic structure calculations. Proc. Natl. Acad. Sci. U.S.A. 2005, 102, 6692–6697. 10.1073/pnas.0408475102. 15845767 PMC1100752

[ref16] Hohenstein E. G.; Sherrill C. D. Density fitting and Cholesky decomposition approximations in symmetry-adapted perturbation theory: Implementation and application to probe the nature of π-π interactions in linear acenes. J. Chem. Phys. 2010, 132, 184111 10.1063/1.3426316.

[ref17] Delesma F. A.; Leucke M.; Golze D.; Rinke P. Benchmarking the accuracy of the separable resolution of the identity approach for correlated methods in the numeric atom-centered orbitals framework. J. Chem. Phys. 2024, 160, 024118 10.1063/5.0184406. 38205851

[ref18] Sun Q. Libcint: An efficient general integral library for Gaussian basis functions. J. Comput. Chem. 2015, 36, 1664–1671. 10.1002/jcc.23981. 26123808

[ref19] Sun Q.; Berkelbach T. C.; Blunt N. S.; Booth G. H.; Guo S.; Li Z.; Liu J.; McClain J. D.; Sayfutyarova E. R.; Sharma S.; et al. PySCF: the Python-based simulations of chemistry framework. WIREs Comput. Mol. Sci. 2018, 8, e1340 10.1002/wcms.1340.

[ref20] Reine S.; Helgaker T.; Lindh R. Multi-electron integrals. WIREs Comput. Mol. Sci. 2012, 2, 290–303. 10.1002/wcms.78.

[ref21] Mai S.; Marquetand P.; González L. Nonadiabatic dynamics: The SHARC approach. WIREs Comput. Mol. Sci. 2018, 8, e1370 10.1002/wcms.1370. PMC6220962 30450129

[ref22] Mai S.; Avagliano D.; Heindl M.; Marquetand P.; Menger M. F. S. J.; Oppel M.; Plasser F.; Polonius S.; Ruckenbauer M.; Shu Y. SHARC3.0: Surface Hopping Including Arbitrary Couplings—Program Package for Non-Adiabatic Dynamics, 2023. Available via the Internet at: https://sharc-md.org/ (accessed February 24, 2025).

[ref23] Schmitt A.; Hinkeldey B.; Wild M.; Jung G. Synthesis of the core compound of the BODIPY dye class: 4,4′-difluoro-4-bora-(3a,4a)-diaza-s-indacene. J. Fluoresc. 2009, 19, 755–758. 10.1007/s10895-008-0446-7. 19067126

[ref24] Chai J. D.; Head-Gordon M. Long-range corrected hybrid density functionals with damped atom-atom dispersion corrections. Phys. Chem. Chem. Phys. 2008, 10, 6615–6620. 10.1039/b810189b. 18989472

[ref25] Papajak E.; Zheng J.; Xu X.; Leverentz H. R.; Truhlar D. G. Perspectives on basis sets beautiful: Seasonal plantings of diffuse basis functions. J. Chem. Theory Comput. 2011, 7, 3027–3034. 10.1021/ct200106a. 26598144

[ref26] Frisch M. J.; Trucks G. W.; Schlegel H. B.; Scuseria G. E.; Robb M. A.; Cheeseman J. R.; Scalmani G.; Barone V.; Petersson G. A.; Nakatsuji H. Gaussian16, Revision C.01; Gaussian, Inc.: Wallingford, CT, 2016.

[ref27] Weigend F.; Köhn A.; Hättig C. Efficient use of the correlation consistent basis sets in resolution of the identity MP2 calculations. J. Chem. Phys. 2002, 116, 3175–3183. 10.1063/1.1445115.

[ref28] Xue B. X.; Barbatti M.; Dral P. O. Machine Learning for Absorption Cross Sections. J. Phys. Chem. A 2020, 124, 7199–7210. 10.1021/acs.jpca.0c05310. 32786977 PMC7511037

[ref29] Zhou Y.; Ni W.; Ma L.; Sun L.; Zhao J.; Gurzadyan G. G. Singlet Fission from Upper Excited States of Bodipy Crystalline Film and Single Crystal. J. Phys. Chem. C 2022, 126, 17212–17222. 10.1021/acs.jpcc.2c05665.

[ref30] Valentini C.; Gowland D.; Bezzu C. G.; Romito D.; Demitri N.; Bonini N.; Bonifazi D. Customising excitation properties of polycyclic aromatic hydrocarbons by rational positional heteroatom doping: the peri-xanthenoxanthene (PXX) case. Chem. Sci. 2022, 13, 6335–6347. 10.1039/D2SC01038K. 35733908 PMC9159094

