# Supplementary material for: Efficient Excitonic Configuration Interaction for Large-Scale Multichromophoric Systems Using the Resolution-of-Identity Approximation
Source: J Phys Chem Lett. 2025 Mar 10;16(11):2800–7. doi: 10.1021/acs.jpclett.5c00065 (PMC11931530; doi:10.1021/acs.jpclett.5c00065)
Supplement: Supplementary file 1 — jz5c00065_si_001.pdf [file jz5c00065_si_001.pdf]

Supporting Information: Efficient Excitonic Configuration Interaction for Large-Scale Multichromophoric Systems Using the Resolution-of-Identity Approximation

Tomislav Piteša,*,†,‡ Sebastian Mai,*,‡ and Leticia Gonzalez*,‡,¶

†*Ruđer Bošković Institute, Bijenička cesta 54, 10000 Zagreb, Croatia.*

‡*Institute of Theoretical Chemistry, Faculty of Chemistry, University of Vienna, Währinger Straße 17, 1090 Vienna, Austria.*

¶*Research Platform on Accelerating Photoreaction Discovery (ViRAPID), University of Vienna, Währinger Straße 17, 1090 Vienna, Austria.*

E-mail: Tomislav.Pitesa@irb.hr; Sebastian.Mai@univie.ac.at; Leticia.Gonzalez@univie.ac.at

S1 ECI basis

Given the ECI expansion and the set of site states, ECI calculations have the following singlet excitonic configurations in the excitonic basis:

$$
\begin{aligned}
{}^1\widetilde{\mathbf{0}} &= |1\mathrm{S}_0, \ldots, M\mathrm{S}_0\rangle\,, && \text{(S1)}\\
{}^1\widetilde{\mathbf{0}}^{F\mathrm{S}_1} &= |1\mathrm{S}_0, \ldots, F\mathrm{S}_1, \ldots, M\mathrm{S}_0\rangle\,, \quad F = 1, \ldots, M, && \text{(S2)}\\
{}^1\widetilde{\mathbf{0}}^{F\mathrm{T}_1, G\mathrm{T}_1} &= \frac{1}{\sqrt{3}}\Big(\big|1\mathrm{S}_0, \ldots, F\mathrm{T}_1^{-1}, \ldots, G\mathrm{T}_1^{1}, \ldots, M\mathrm{S}_0\big\rangle \\
&\quad - \big|1\mathrm{S}_0, \ldots, F\mathrm{T}_1^{0}, \ldots, G\mathrm{T}_1^{0}, \ldots, M\mathrm{S}_0\big\rangle \\
&\quad + \big|1\mathrm{S}_0, \ldots, F\mathrm{T}_1^{1}, \ldots, G\mathrm{T}_1^{-1}, \ldots, M\mathrm{S}_0\big\rangle \Big), \\
&\quad F, G = 1, \ldots M; \quad F < G, && \text{(S3)}\\
{}^1\widetilde{\mathbf{0}}^{F\mathrm{S}_1, G\mathrm{S}_1} &= |1\mathrm{S}_0, \ldots, F\mathrm{S}_1, \ldots, G\mathrm{S}_1, \ldots, M\mathrm{S}_0\rangle\,, \\
&\quad F, G = 1, \ldots M; \quad F < G, && \text{(S4)}
\end{aligned}
$$

The first configuration is the ground-state (GS) product, the second ones are M different S_1 LEs, the third one are $\binom{M}{2}$ different T_1-T_1 DLEs, and the last ones are $\binom{M}{2}$ different S_1-S_1 DLEs.

Triplet configurations present in the ECISD basis

$$
\begin{aligned}
{}^3\widetilde{\mathbf{0}}^{F\mathrm{T}_1} &= \big|1\mathrm{S}_0, \ldots, F\mathrm{T}_1^{1}, \ldots, M\mathrm{S}_0\big\rangle\,, \quad F = 1, \ldots, M, && \text{(S5)}\\
{}^3\widetilde{\mathbf{0}}^{F\mathrm{T}_1, G\mathrm{T}_1} &= \frac{1}{\sqrt{2}}\Big(\big|1\mathrm{S}_0, \ldots, F\mathrm{T}_1^{0}, \ldots, G\mathrm{T}_1^{1}, \ldots, M\mathrm{S}_0\big\rangle \\
&\quad - \big|1\mathrm{S}_0, \ldots, F\mathrm{T}_1^{1}, \ldots, G\mathrm{T}_1^{0}, \ldots, M\mathrm{S}_0\big\rangle \Big) \\
&\quad F, G = 1, \ldots M; \quad F < G, && \text{(S6)}\\
{}^3\widetilde{\mathbf{0}}^{F\mathrm{T}_1, G\mathrm{S}_1} &= \big|1\mathrm{S}_0, \ldots, F\mathrm{T}_1^{1}, \ldots, G\mathrm{S}_1, \ldots, M\mathrm{S}_0\big\rangle\,, \\
&\quad F, G = 1, \ldots M; \quad F \neq G, && \text{(S7)}
\end{aligned}
$$

are, respectively, M different T_1 LEs, $\binom{M}{2}$ different T_1-T_1 DLEs, and $M(M-1)$ different T_1-S_1 DLEs. For triplet site states, m_S values are denoted as superscript. Note that the direct TD-ωB97XD calculation can intrinsically describe only ${}^1\widetilde{\mathbf{0}}^{F\mathrm{S}_1}$ and ${}^3\widetilde{\mathbf{0}}^{F\mathrm{T}_1}$ configurations, due to CIS-like nature of linear-response TD-DFT. Hence, we compare only the states dominantly described by LEs between (RI-)ECISD and the direct calculation, and merely present the behaviour of the dominantly DLE states obtained in (RI-)ECISD calculations.

S2 Spectrum and Density-of-States Calculations

Absorption cross section and DOS are calculated by Gaussian broadening of the line contributions, i.e.,

$$\sigma(E) = \frac{1}{M}\frac{\pi\hbar e^2}{2m_e c\varepsilon_0 E}\sum_I E_{\mathrm{ex},I} f_{I0} G_I(E) \tag{S8}$$

$$\mathrm{DOS}(E) = \sum_I G_I(E), \tag{S9}$$

where sum per I goes over excited full-system states, while $E_{\mathrm{ex},I}$ and f_{I0} are excitation energy and oscillator strength of the excited state I, and where normalized Gaussian-broadening function for each line signal reads

$$G_I(E) = \frac{1}{\delta E}\sqrt{\frac{2}{\pi}}\exp\left\{-\frac{\left(E - E_{\mathrm{ex},I}\right)^2}{2\left(\delta E/2\right)^2}\right\}. \tag{S10}$$

Parameter δE defines the width of the broadening function, and the used value is given in the caption of each figure in the main text for the respective system. As can be seen, cross sections were normalized to the number of fragments M, to obtain a quantity proportional to the molar extinction coefficient.

S3 Error Analysis

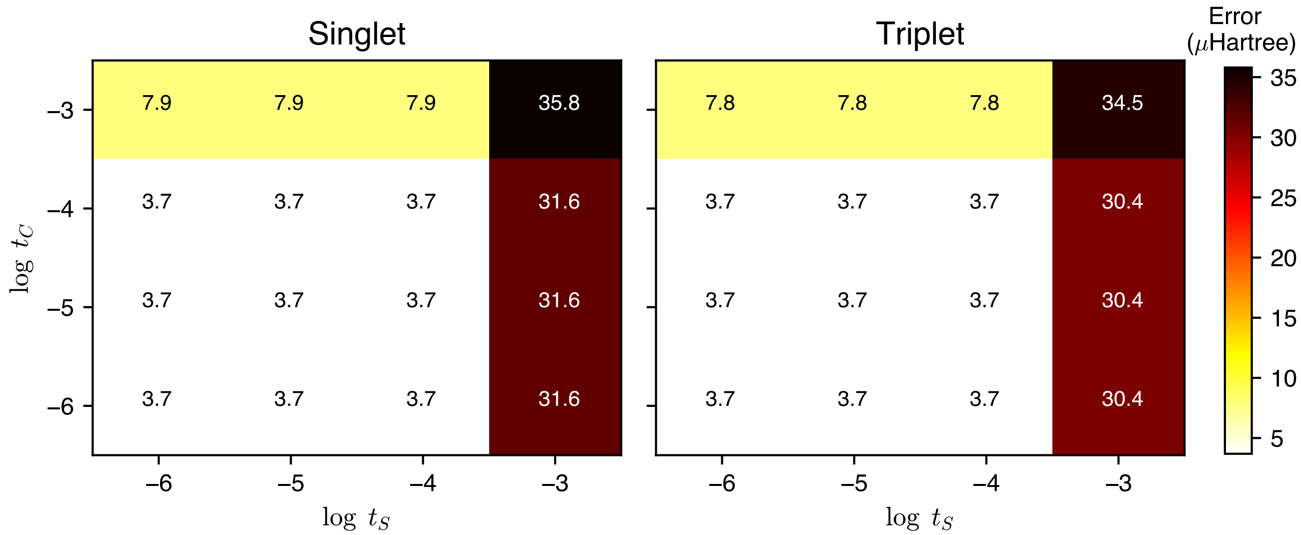

Figure S1: Greatest absolute value of K terms in RI-ECISD with respect to ECISD calculation of BODIPY dimer ($M = 2$) for different values of t_S and t_C thresholds, for singlet and triplet ECI Hamiltonian separately.

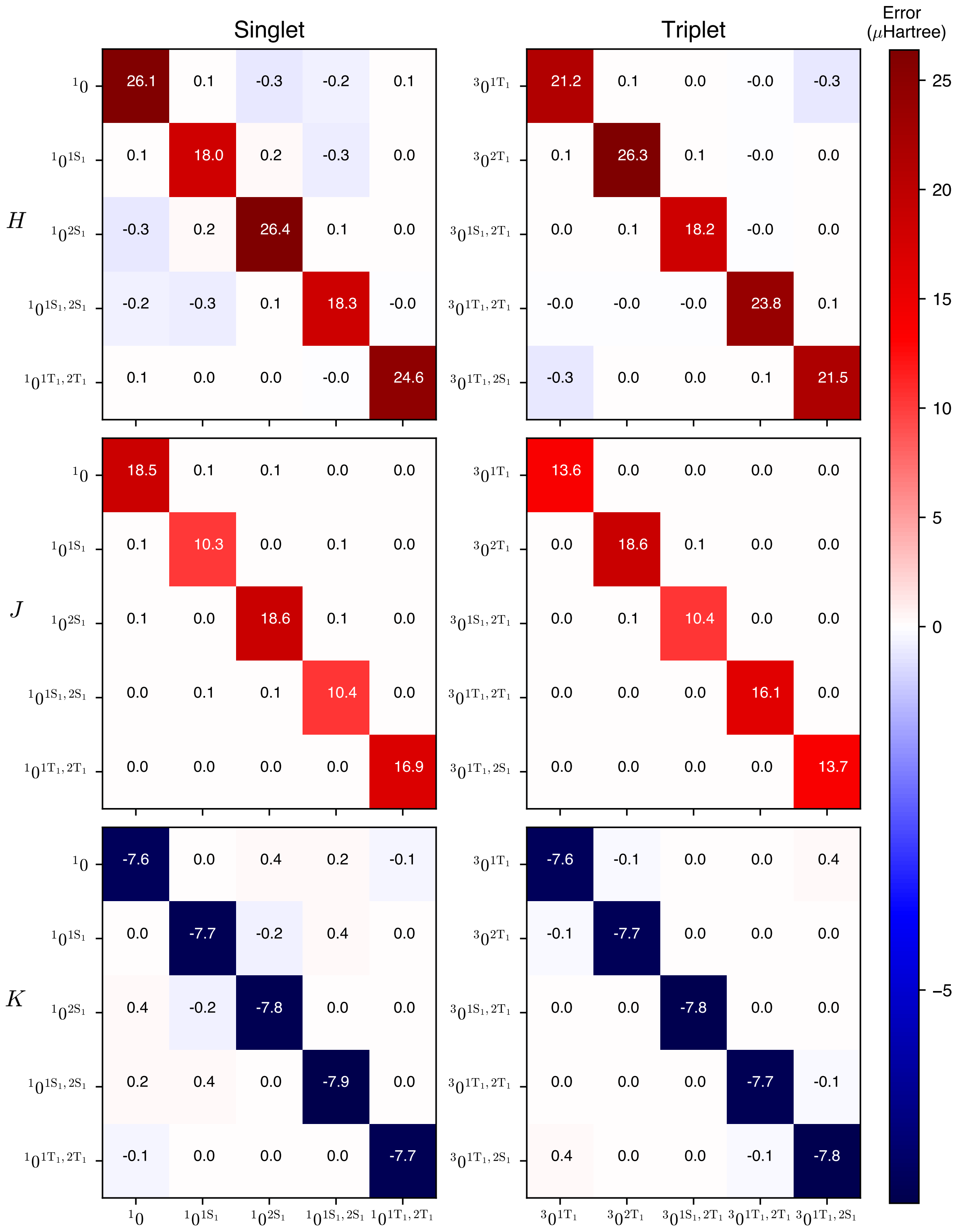

Figure S2: Greatest absolute value of K terms in RI-ECISD with respect to ECISD calculation of BODIPY dimer ($M = 2$) for different values of t_S and t_C thresholds, for singlet and triplet ECI Hamiltonian separately.

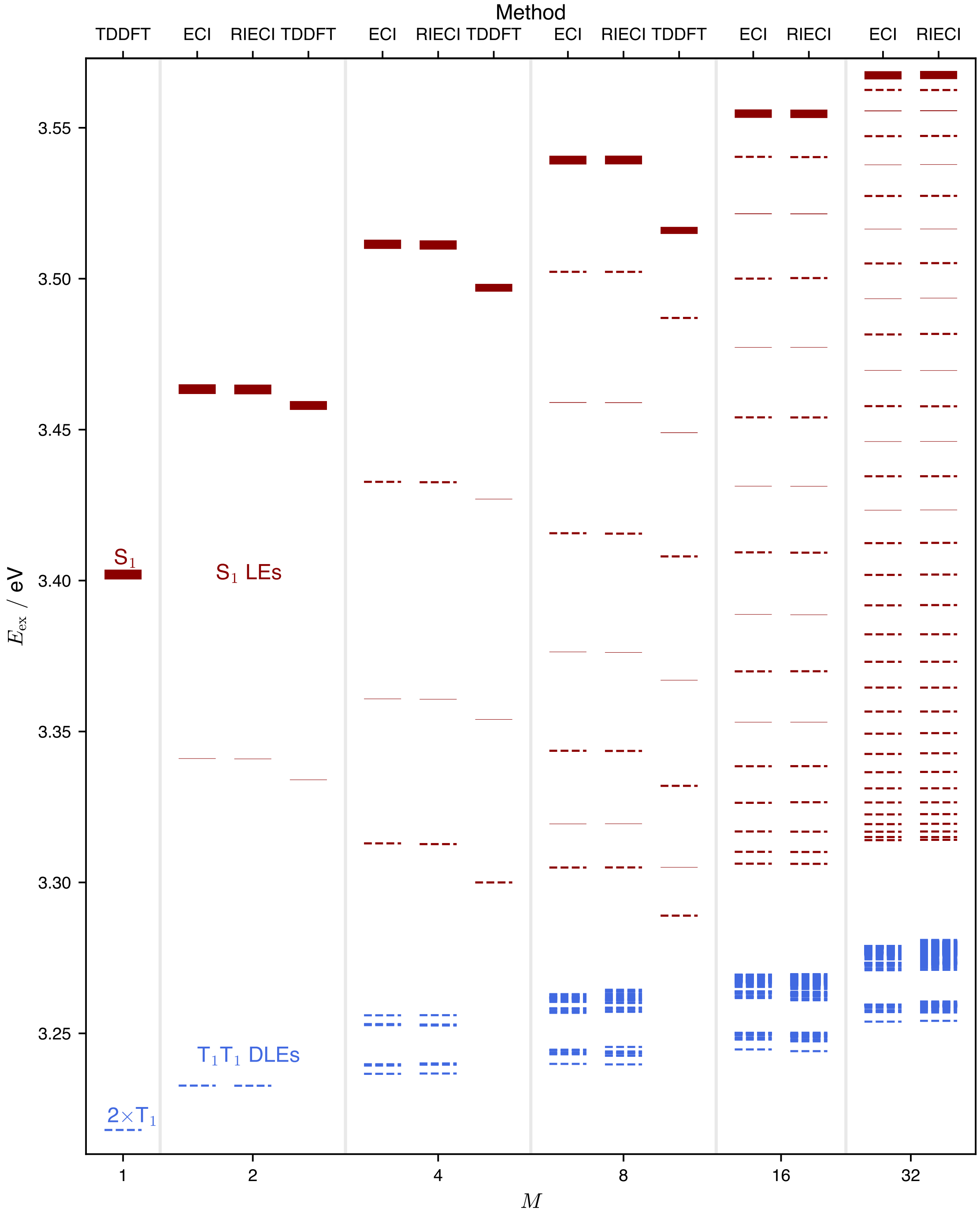

Figure S3: Excitation energies of the singlet states dominantly spanned S_1 LEs (red) and T_1T_1 DLEs (blue) of the chains containing M BODIPY molecules (lower x-axis), calculated with direct TDDFT calculation, ECISD and RI-ECISD (upper x-axis). The thickness of the line is proportional to the oscillator strength of the respective state, while the dashed lines represent the states with oscillator strength lower than 0.01. For $M = 1$, two lines represent the T_1 state on doublet excitation energy and the S_1 state (dashed blue and full red respectively).

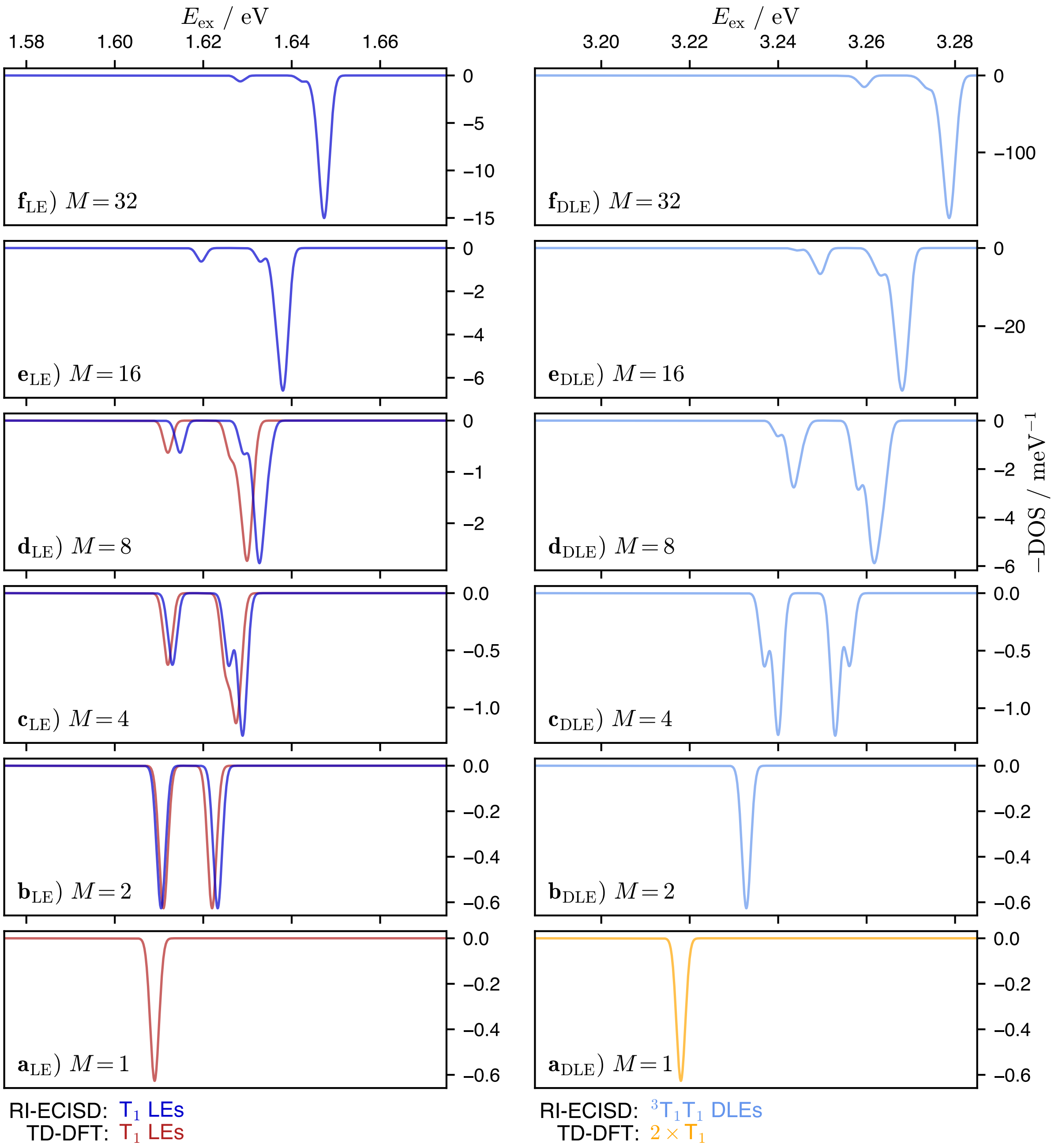

Figure S4: Density of states (DOS) of the T_1 LEs (left panels) and triplet T_1T_1 DLEs (right panels) for the segments of BODIPY chain containing $M = 1, 2, 4, 8, 16, 32$ fragments. DOS of LEs calculated by a direct TD-DFT calculation are shown in red and by RI-ECISD in dark blue, while the DOS of DLEs calculated by RI-ECISD are plotted in light blue. Orange curve for $M = 1$ represent the position of doubled excitation energy of the T_1 site state. All DOS on this figure are calculated by Equation S9 with $\delta E = 2$ meV.
